# Bioremediation of cadmium using cadmium-tolerant bacteria in cacao farms in Arauca, Colombia

**DOI:** 10.1007/s13205-026-04790-3

**Published:** 2026-04-23

**Authors:** Laura Alvarez-Rubiano, Pedro Brito Brandão, Daniel Bravo

**Affiliations:** 1 Facultad de Ciencias Agropecuarias, Universidad Nacional de Colombia - Sede Palmira, Carrera 32 # 12 - 00, Valle del Cauca, Palmira, Colombia; 2 Facultad de Ciencias, Departamento de Química, Universidad Nacional de Colombia – sede Bogotá, Avenida Carrera 30 #45-3, Bogotá, Cundinamarca Colombia; 3 Laboratory of Soil Microbiology & Calorimetry, Corporación Colombiana de Investigación Agropecuaria AGROSAVIA. Research Centre CI Tibaitatá, Km 14 Vía Bogotá - Mosquera, Mosquera, Cundinamarca 20040 Colombia

**Keywords:** *Theobroma cacao* L., Isothermal microcalorimetry, Droplet digital PCR, Cd immobilization

## Abstract

A mitigation strategy involving the application of cadmium-tolerant bacteria (CdtB) was assessed in cacao soils in farms in Arauca, Colombia. The bacterial strain used, *Pseudomonas chlororaphis* CdtBSO has exhibited an ability to immobilise cadmium, reducing its bioavailability to cacao. The aim of this study was to increase the immobilization activity of CdtB in cacao soils in Arauca. Five treatments combining different concentrations of CdtB with a synthetic fertiliser (Agrocacao) and zeolite were established. The *cadA* gene as marker for Cd immobilization was detected using droplet digital PCR (ddPCR). Moreover, the metabolic activity of CdtB was assessed using isothermal microcalorimetry (IMC). Maximum thermal performance was observed in the treatments where the most CdtB was applied. However, the results were highly dependent on the site. An increase in soil Cd content six months after application of the treatments was observed suggesting increased immobilization rates of the metal in the soil. The explanation of possible immobilisation mechanisms varied between treatments. However, the treatment showing the most positive trends included 50% CdtB and 50% Agrocacao. The detection of the *cadA* gene, revealed variations in the relative abundance of the copies of the gene in the assessed soils. This work contributes to the knowledge on soil Cd bioremediation using CdtB, through the application of innovative approaches to monitoring, such as ddPCR and IMC. It enriches the information available on the application of physicochemical and biological amendments for the remediation of Cd in real cacao crop conditions.

## Introduction

Colombia is the third largest cacao bean producer in Latin America, with production reaching 59,831 Mg in 2025 (ICCO 2025). The focus of this paper is the region of Arauca which is the second largest producer of cacao, contributing 12.7% to the total (FEDECACAO 2025).

Cadmium (Cd) is a heavy metal and is distributed throughout the earth’s crust, entering the environment naturally mainly through the weathering of parent material (Alloway 2013), and to a minor extent through the use of contaminated fertilisers (López-Zuleta et al. 2025). It is worth mentioning that the cacao tree (*Theobroma cacao* L.) has an ability to accumulate cadmium (Chavez et al. 2015), which is absorbed, translocated and accumulated mostly in the leaves, with a lower concentration in fruit pods and beans. This has resulted in series of challenges for producers, due to food safety regulations established by overseas markets that must be met (European-Commission 2014). In particular, the regulation 488/2014 established by the European Union (EU) through the European Food Safety Authority (European-Commission 2014), specifies maximum permissible limits for cadmium content in chocolate and cacao products ranging from 0.1–0.8 mg kg^−1^ Cd depending on the percentage of cacao mass in the final product. This regulation arises from the concern about the presence of cadmium in food and its effect on human health’s.

This has led to the search for solutions to reduce uptake of Cd by cacao through soil remediation. These include, experiments involving the application of chemical fertilisers such as Agrocacao (a synthetic fertiliser) and mineral amendments such as zeolites and liming, in Ecuador, Trinidad and Tobago (Ramtahal et al. 2016; Argüello et al. 2019) and Colombia (Bravo et al. 2021). Agrocacao is a balanced source of minerals required for cacao nutrition, increasing yields and reducing loss due to nutrient deficiencies. Zeolite has been suggested to be useful due to its high cation-exchange capacity so it can bind ions such as cadmium in the soil. However, the results with zeolite have not been particularly convincing and there is a need for other solutions.

Interestingly, very few studies have explored the use and metabolic capacity of soil microorganisms with immobilization activity (Bravo et al. 2018; Adarme-Duran et al. 2024). In this context, cadmium-tolerant bacteria (CdtB) play a key role in the transformation of the metal, facilitating its biotransformation through specific metabolic processes (such as bioleaching, biotransformation or bioweathering) due to the activation of genes (such as *cadA*) that use cadmium for P-type ATP-dependant proteins involved in Cd membrane efflux pumps (Mergeay 1997). These mechanisms lead to modifications in the Cd binding with other elements, reducing its mobility and favouring its conversion into insoluble or inactive geostable compounds, such as otavite (CdCO_3_), greenockite (CdS) or hawleyite (CdS) (Bravo and Braissant 2022). This decreases Cd bioavailability and thus reduces uptake by plants (Dai et al. 2019; Bravo and Braissant 2022).

While the expression of the *cadA* gene in bacterial populations does not demonstrate immobilisation of cadmium in the soil, it may indicate the physiological potential of bacteria to tolerate it and survive in contaminated environments, since the protein is part of the metabolic machinery of metal bioweathering and bioleaching pathways, as explained in previous studies (Mergeay 1997; Naz et al. 2005; Bravo & Braissant 2022). The digital droplet PCR technique (ddPCR) allows detection of this gene in a soil sample and can used as an indicator that these bacteria are present, although it cannot provide information on their activity to immobilise Cd. To quantify this, other molecular or thermodynamic methods are required, such as bromodeoxyuridine DNA (BrdU) labelling (Bravo et al. 2013) or isothermal calorimetry (IMC) (Bravo et al. 2011; Braissant et al. 2013), respectively. Thus, the presence of the *cadA* gene should be interpreted as a marker of tolerance, and a very useful indicator of potential metal sequestration through geochemical stabilisation.

In Arauca the high cadmium concentrations in cacao have been shown to be due to the sedimentation from the Arauca River, with a north–south trend of decreasing soil Cd concentration from Arauquita and Saravena, to Fortul and Tame (Bravo et al. 2024a, b). However, until now, no studies have focused on mitigation strategies, particularly using microorganisms as a bioremediation process to tackle the problem. The hypothesis is that, using CdtB, geochemically stabilised Cd will increase in soils due to the immobilization activity of the bacteria. Thus, the aim of this research was to determine the effect of the application of a CdtB characterised strain on soil cadmium immobilization in cacao farms in Arauca. Soil Cd content was measured using monochromatic energy dispersive X-ray fluorescence MXRF (E-MAX, NY, USA), recently validated (Cifuentes et al. 2025), and analysing the bacterial metabolic activity, using IMC and its relationship with the presence of the gene *cadA* measured using ddPCR.

## Materials & methods

### Study area and farm selection

The study was carried out on four farms, with one farm in each of the four municipalities in the Arauca department. The municipalities were: Arauquita, Saravena, Fortul and Tame and represents the principal cacao growing areas within the department (Bravo et al. 2024a, b). Further information about the samples is given in Table 1. Arauquita (AR_1) is to the north and forms part of the Orinoco river basin; to the northwest, Saravena (SA_2), which includes the eastern slopes of the Andean foothills, Fortul (FO_3), includes mountainous territories to the west and slightly undulating to flat lands to the east; and in the southwest of Arauca is Tame (TA_4), which includes mountainous territories to the east, where two different topographic regions can be distinguished, i. a mountainous region to the west corresponding to the eastern slope of the eastern mountain range and ii. another slightly undulating or flat region to the east (Gómez-Tapias et al. 2017). The farms soil properties and agricultural practices are well studied (Bravo et al. 2024a, b) and were selected for having some of the highest cacao yields in the department, similar topographical features and medium to high bean cadmium concentrations.

**Table 1:** The treatments were applied to farms in four municipalities in the Arauca department (Arauquita—AR_1; Saravena—SA_2; Fortul—FO_3; and Tame—TA_4)

Date of application	ID Farm	Treatment	CdtB (1 × 10^8^ CFU) [mL]	Zeolite [g]	Agrocacao [g]
March 13—2023	AR_1	T3	62.5	125	750
March 14—2023	SA_2	T5	62.5	–	750
March 15—2023	FO_3	T7	125	500	250
March 16—2023	TA_4	T8	250	–	500
March 13- 2023	All	T9	Blank		

The treatments were applied as a combination of sources, with the dose per tree varying according to the concentrations of Zeolite and Agrocacao. The different concentrations of each compound prevented one compound from predominating over the other

### Treatments application and collection of samples

The 2 ha study areas were selected as the most representative of each farm, with tree age, ranging from 5 to 7 years. Five treatments were established per farm, using a combination of CdtB, zeolite as a chelating mineral amendment (Zeocol S.A.S) and a synthetic chemical fertiliser (Agrocacao), to providing nitrogen. The treatments were as shown in Table 1. The chemical composition and Cd concentration of Agrocacao and zeolite are shown in Tables S1 and S2. A mass balance calculation was not required as the Cd content in the amendments was lower than that found in the soil. A recent study (López-Zuleta et al. 2025), confirmed that the amount of mobile Cd fractions (water-soluble and exchangeable) was very low for Agrocacao and zeolite. Therefore, the amount of Cd that could enter the cacao system from these amendments in this experiment is negligible. Each experimental unit (block) was approximately 100 m^2^. Five experimental units were distributed in each farm, each comprising of 9 trees. The units were comprised by three replicates per treatment. Composite soil samples were collected from 3 holes 70 cm from each tree trunk at a depth soil of 20 cm. This corresponds to the area of high root density. Composite leaf samples were also collected, once for a baseline at the beginning of the experiment and at the end after six months, per municipality.

The CdtB used was a *Pseudomonas chlororaphis* strain CdtB-SO that was previously isolated from cacao farms in Colombia (Bravo et al. 2018). It forms part of a bioproduct developed by AGROSAVIA (The Colombian Research Centre for Agriculture) for soil application in Cd hotspots at a national level (Bravo et al. 2021). In vitro and greenhouse experiments performed with this strain have demonstrated a great capacity for Cd immobilisation of up to 12 mg kg^−1^ Cd 12 days after inoculation (Bravo et al. 2018). It is worth mentioning that the strain poses no biosafety or hazardous risks confirmed by previous experiments performed by AGROSAVIA (unpublished). The CdtB bacterial strain was applied at a concentration of 1 × 10^8^ CFU mL^−1^ on each farm together with the Agrocacao and zeolite, according to the doses proposed in each of the treatments (see Table 1).

The samples were homogenised to obtain approximately 200 g of differentiated soil for each of the treatments and blocks, which were placed in zip lock bags together with detailed information including the municipality where it was collected, name of the farm, which treatment, together with the block number and date of collection. The samples were transported and stored at 4 °C for conservation at the Calorimetry and Genetics Laboratories, in the Research Centre Tibaitatá AGROSAVIA, in Mosquera, Colombia, for further processing of both thermodynamic and molecular analysis.

### Cadmium determination

The monochromatic energy dispersive X-ray fluorescence MXRF (E-MAX, NY, USA) technique was used to quantify the concentration of Cd in soils (Lewis et al. 2024). For the analysis of cadmium in the samples, the soil was previously oven-dried and sieved with a 0.2 µm sieve. The MXRF sample cup was covered at the top with a 12 µm polypropylene film (reference: PP Film "Wide X91, 4 m Z-spec Inc. NY, USA), and approximately 1 g of soil was placed inside the sample cup and compacted with a plunger. The ‘soil’ mode calibration (*y* = 2813.13 *x* + 35.59) was used for soil samples, where ‘*y*’ represents the counts per second (CPS) and ‘*x*’ constitutes the concentration (Cifuentes et al. 2025). Three measurements were performed on each sample, rotating the sample between each measurement and a mean was calculated. As quality controls for Cd testing, duplicates and certified reference materials (CRMs) of soil and leaf were used. The CRMs were NIST® 2709a San Joaquin Soil and 1570a Spinach leaves. The CRMs were included every 10 samples to assure metrological quality. For Cd measurement of CdtB in LB liquid culture media, the method validated for E-max (Cifuentes et al. 2025) was used. For this analytical technique, the detection limit was determined using the lower limit of precision as reported by (Cifuentes et al. 2025). This was calculated by multiplying the standard deviation of the cadmium content in each farm studied by 3. Two subsamples were analysed for each soil sample (n = 2). This method achieved a limit of quantification (LOQ) of 0.148 mg kg^−1^, with recovery percentages ranging from 90.7 to 109.0% and *Z*’ scores for reproducibility between 0.50 and 0.62 (Cifuentes et al. 2025). The distribution of pseudo-total cadmium in the studied farms was determined from soil samples collected at two points in time: before (t_0_) and after (t_1_) the application of the treatments.

Furthermore, the available fraction of Cd in soils was quantified with CaCl_2_, according to previous studies (Rinklebe & Shaheen 2014; Rinklebe et al. 2016; López-Zuleta et al. 2025) by ICP-MS, and Cd in cacao leaves was quantified according to a previous study (Bravo et al. 2024a, b), using MXRF. Four average measurements were performed to determine the available soil Cd and leaf Cd content for each municipality studied.

For both molecular and thermodynamic analyses, soil samples were processed as random samples (representative samples composed by treatment), therefore, the results describe average biological trends of the treatment.

### Bacterial soil DNA extraction

For the extraction and purification of genomic DNA (gDNA) from soil samples, the DNeasy® Powersoil® kit was used (Bravo et al. 2015), following the protocol suggested by the manufacturer QIAGEN®. The gDNA obtained was stored at −20 °C until analysis.

### Amplification of the *cadA* gene as a proxy for CdtB detection using ddPCR

In soil samples, amplification of the *cadA* gene, associated with cadmium tolerance, was performed. This gene is present in cadmium-tolerant bacterial strains. The primers used in this process were previously by (Bravo 2022). The specific primers were designed based on the reported gene sequence for the species *Pseudomonas chlororaphis*. The sequences used were: Fw (forward) 5'-GCCGGATAATCATCGAGAACCT-3' and Rv (reverse) 5'-CACCACGGTCAACGAACTGAT-3' (Naz et al. 2005; Singh et al. 2020; Bravo and Braissant 2022), with an expected amplicon size of 131 bp. The primers specificity was verified through a BLAST analysis with the chromosome sequence of the complete genome of *Pseudomonas chlororaphis* strain qlu-1, available in GenBank with the NCBI Reference Sequence: NZ_CP061079.1. In this study, we designed ddPCR primers (software LGC Biosearch technologies) using genomes found in GenBank to identify the conserved regions reported for *cadA* in the *Pseudomonas* genus. Then, an *in-silico* evaluation was performed. It was used BLASTn to screen the primer sequences against a collection of bacterial genomes from *Pseudomonas* genus. We confirmed that the primers matched the target genus sequences and did not match non-target groups. Once the primers had been synthesised, we performed target panel testing by running ddPCR with genomic DNA from *P. chlororaphis* and *P. oleovorans*. A positive signal in each of the expected target strains confirmed the target coverage proposed for this study. For the ddPCR assays, a TaqMan probe labelled with the FAM fluorophore was used, with the following sequence: 5'-CCGGGCTGTACAATGGCCTCA-3'. It is important to note that, to assess the persistence of the strain included after soil application, the probes designed for the primers studied here were specifically constructed for *P. chlororaphis* (software LGC Biosearch technologies), as the active ingredient of the bioproduct being developed by AGROSAVIA.

To verify the correct amplification of the fragment and establish the limit of detection (LOD) of the assay, a g-Block was used, corresponding to a synthetic double-stranded DNA sequence that includes the *cadA* gene region with the binding sites of the primers and probe described above. This synthetic material was used exclusively as a positive control and reference material for standardising the method. Serial dilutions were prepared from the g-Block in nuclease-free water, covering a range of decreasing concentrations. Each dilution was analysed using the same amplification assay configuration to determine the detection sensitivity. The results showed that the minimum reproducibly detectable concentration was 50 femtograms (fg) of g-Block DNA, establishing a value of 1.41 × 10^2^ copies of the *cadA* gene per gram of soil (copies g soil⁻^1^) as the limit of detection and 2.7 × 10^3^ copies g soil⁻^1^, as the limit of quantification for the *cadA* gene, according to the experimental conditions used in this study.

DNA samples were quantified by spectrophotometry using a NanoDrop™ device (Thermo Fisher Scientific, USA) to determine the concentration and purity of the total DNA obtained. Based on the initial concentrations, each sample was adjusted to a uniform concentration of 5 ng µL^−1^ by diluting with nuclease-free nanopure water to a final volume of 20 µL. This normalisation allowed the same amount of DNA to be used in the PCR reactions, ensuring comparability between treatments (See supplementary Figure S1). In each PCR reaction, 5 µL of DNA was used, equivalent to 25 ng of total DNA from 1 g of a composite soil sample, from which the genetic material was extracted. Therefore, the amplification results are presented as copies g soil⁻^1^ of the gene *cadA*.

A final reaction volume of 24 µL was prepared with a super mix for probes consisting of: i. no dUTP 2 × (Bio-Rad), ii. a kit of FAM—HEX 100 Um (micromolar) labelled probes (Lovera et al. 2024), iii. the forward and reverse 100 Um primers, and iv. the extracted and purified soil DNA. Besides, 20 µL of the ddPCR mix were transferred into the sample wells of the Bio-Rad cartridge (Bio-Rad Laboratories, Inc. CA, USA) and 70 µL of droplet-generating oil into the respective oil wells, where droplets were formed in the QX-200 droplet generator (Bio-Rad Laboratories, Inc. CA, USA). From this resulting emulsion, 40 µL were transferred into a 96-well PCR plate and sealed using the PX1TM plate sealer device of the ddPCR system (Bio-Rad Laboratories, Inc. CA, USA). The sealed plate was placed in the T100TM deep well contact thermal cycler (Bio-Rad Laboratories, Inc. CA, USA). The ddPCR conditions were: 10 min at 95 °C; followed by 40 cycles of 94 °C for 30 s and 60 °C for 60 s; and at the end of the reaction, a 10 min hold at 98 °C and cooling to 4 °C, with a temperature ramp of 2 °C each s. After the ddPCR copies were obtained, the plate was placed in the QX200 droplet reader (Bio-Rad), where the data were analysed with the Quanta Soft Analysis software (Bio-Rad Laboratories, Inc. CA, USA). To establish the difference between positive and negative samples, a threshold was set, which is determined with the droplets found at the upper limit of the NTC as shown in a previous study (Lovera et al. 2024). This was done by applying an amplitude threshold set just above the amplitude signal corresponding to negative droplets. Consequently, samples whose concentration values were less than or equal to the cut-off value defined by the negative control were classified as negative.

#### Metabolic activity of CdtB assessed by isothermal microcalorimetry (IMC)

The measurements of heat-flow produced by the microbial metabolic activity were performed to explore trends, in an air conduction isothermal microcalorimeter (TAM Air, Waters/TA Instruments, Delaware, USA) equipped with 8-channel configuration. Heat-flow was measured in Watts, and the heat was estimated in Joules. Kinetic interpretations of maximum growth rate and time to the peak (that corresponds to the metabolite production rates) were obtained from mathematical integration of heat (Braissant et al. 2004; Bravo et al. 2011).

Microbial metabolism was analysed using the IMC technique (Bravo et al. 2018; Bravo and Braissant 2022). With this technique is possible to determine time-dependent changes in heat-flow produced by the metabolic activity of bacteria, particularly when the microorganism is growing in a cadmium-selective medium. To achieve this, a Mergeay liquid media enriched with CdCl_2_ (Sigma Aldrich, USA) was used (Bravo et al. 2018).

The soil was sieved with a 1.18 µm sieve. The soil samples were homogenised removing large particles and plant material such as roots. One gram of soil was placed in a sterile 20 mL plastic ampoule. The ampoules contained 10 mL of Mergeay liquid medium (Mergeay 1995) and 10 mg L^−1^ of glucose.

The use of Mergeay media is critical to define Cd-tolerance. Since Cd may interact with many components of other growth media, especially with phosphates, broth components and with organic acids used as carbon sources, the Mergeay minimal growth media is highly recommended. The Mergeay media (Mergeay 1995) was designed to mimic sediments from polluted environments which may contain up to 10% of heavy metals but is still able to provide a habitat for viable heterotrophic bacteria. Thus, this minimal media is required to avoid complexation of Cd with other components and improve the Cd immobilization assessment with soil Cd-tolerant bacteria (Bravo 2022), or with isolated CdtB strains (Bravo et al. 2018) or with artificially enhanced populations, such as is the case here. The Mergeay medium has been used to assess high binding capacity for cadmium (Lata et al. 2023), and specifically for testing bacterial Cd stress (Knotek-Smith et al. 2003). The Mergeay media has been also used for several studies using techniques such as XRF to assess the immobilization capacity of the isolated strains such as *Stenotrophomonas*, *Achromobacter* and *Staphylococcus* spp. (Fan et al. 2024), analysing metallophilic, metal-resistant, and metal-tolerant microorganisms (Kanekar and Kanekar 2022).

To increase immobilization activity to the cadmium assays, the same amount of sample was amended with 10 mL of Mergeay liquid medium with 6 mg L^−1^ of CdCl_2_ (Sigma Aldrich, USA) and 10 mg L^−1^ of glucose (Bravo 2022). Before placing each vial in the channels of the TAM Air, reference vials containing sterile Mergeay medium were added to the reference side. The calorimetric assays were performed with a thermal equilibrium at 25 °C providing thermostable conditions for metabolic activity. The experiment was performed in 30 h at isothermal conditions.

To differentiate the microbial heat flow from abiotic reactions, a baseline was performed with blanks. The baseline was performed with ampoules containing sterile Mergeay media with the same Cd concentrations as the inoculated ampoules. Therefore, the microbial metabolic activity was recorded by the automatic subtraction of the chemical reactions of the ampoules used as blank with the measured ampoules with the treatments. The measurements performed in the TAM Air were recorded after the recommended two-step thermal equilibration procedure (Braissant et al. 2010; Bravo et al. 2011) that lasted 1 h.

### Data analysis

To perform a simple comparison, multifactorial ANOVA variance analysis was included using GLM (generalised lineal model) procedures (Yang et al. 2022; Salinas Ruíz et al. 2023). The following were considered fixed factors in the model: i. municipality, corresponding to the different sampling farms; ii. treatment applied, with five levels; and iii. treatment application time. Likewise, the ‘municipality × treatment’ interaction was included to identify possible combined effects between geographical location and the treatments applied. The dependent variable analysed corresponds to the actual cadmium content (mg kg^−1^) in soil measured experimentally. The statistical analysis was performed at a significance level of 5% (p < 0.05). This analysis was performed using SAS software (v. SAS enterprise guide 8.3, 2026). Moreover, since average values of available Cd in soils and Cd in leaves were obtained per municipality, a one-sample t-test was performed to compare the difference between the initial and the final available Cd content in soils, or Cd in leaves, in the four municipalities, and test whether there was a reduction relative to the initial value. The statistical analysis was performed at a significance level of 5% (p < 0.05). This analysis was performed using QtiPlot software (v. Qt 5.12.8). The obtained thermograms were analysed using R software and the thermodynamic and kinetical growth conditions were statistically analysed with mean comparation test. Moreover, the thermograms were used to discuss the exploratory thermodynamic signatures of microbial activity, and a simple linear correlation was performed between heat flow and the time to arrive to the pick of maximal metabolic activity (TTP). It is worth mentioning that all molecular and calorimetric results are framed as qualitative or exploratory trends because the samples used corresponded to composite samples and not independent biological replications.

## Results and discussion

### Cadmium distribution in soils

**Fig. 1:**
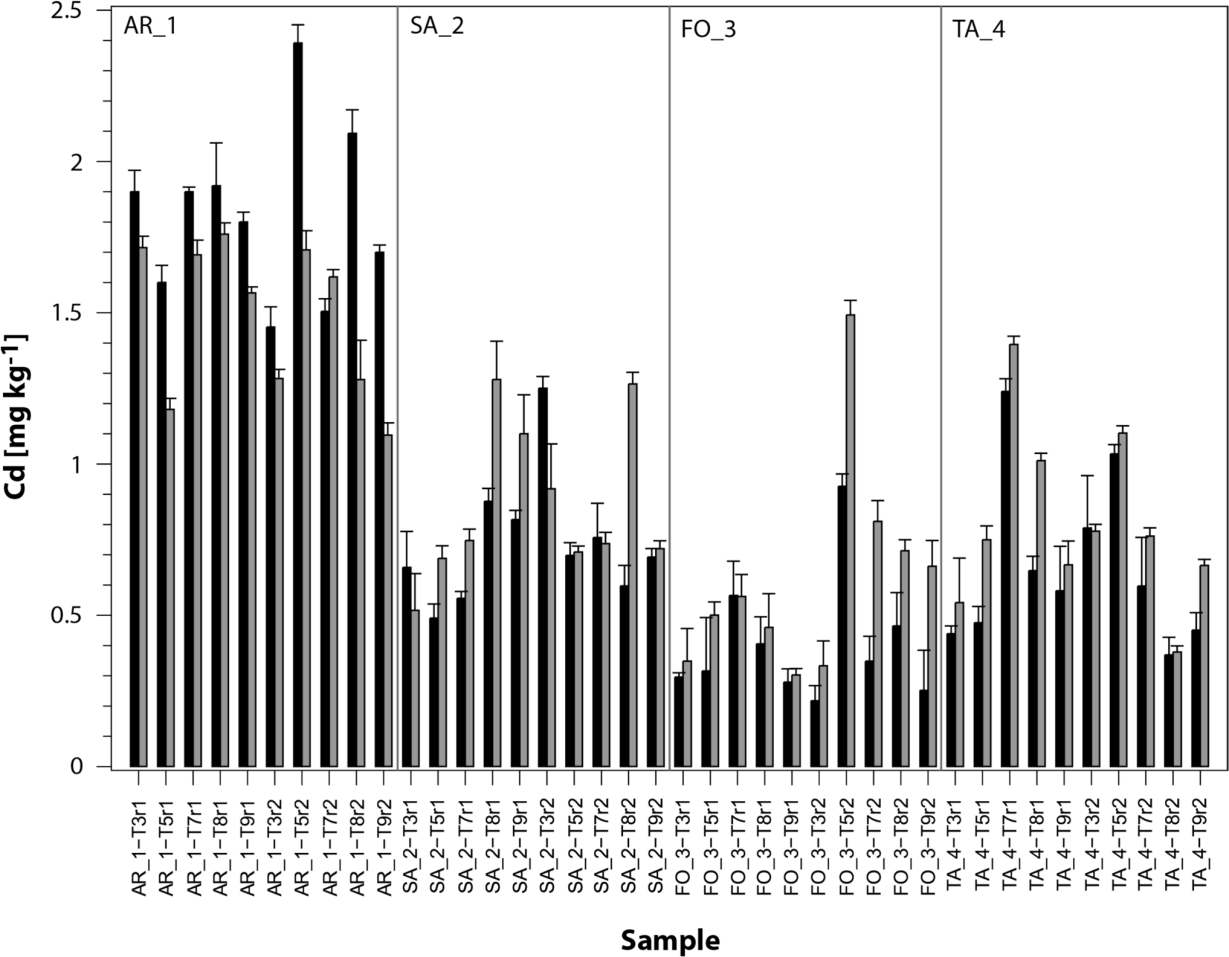
Soil cadmium content in all the treatments performed. The black bars show the Cd content before (t_0_) applications. The grey bars indicate Cd content after (t_1_) the application of the treatments (T). The vertical bars corresponds to the error bar (n = 2)

**Fig. 2:**
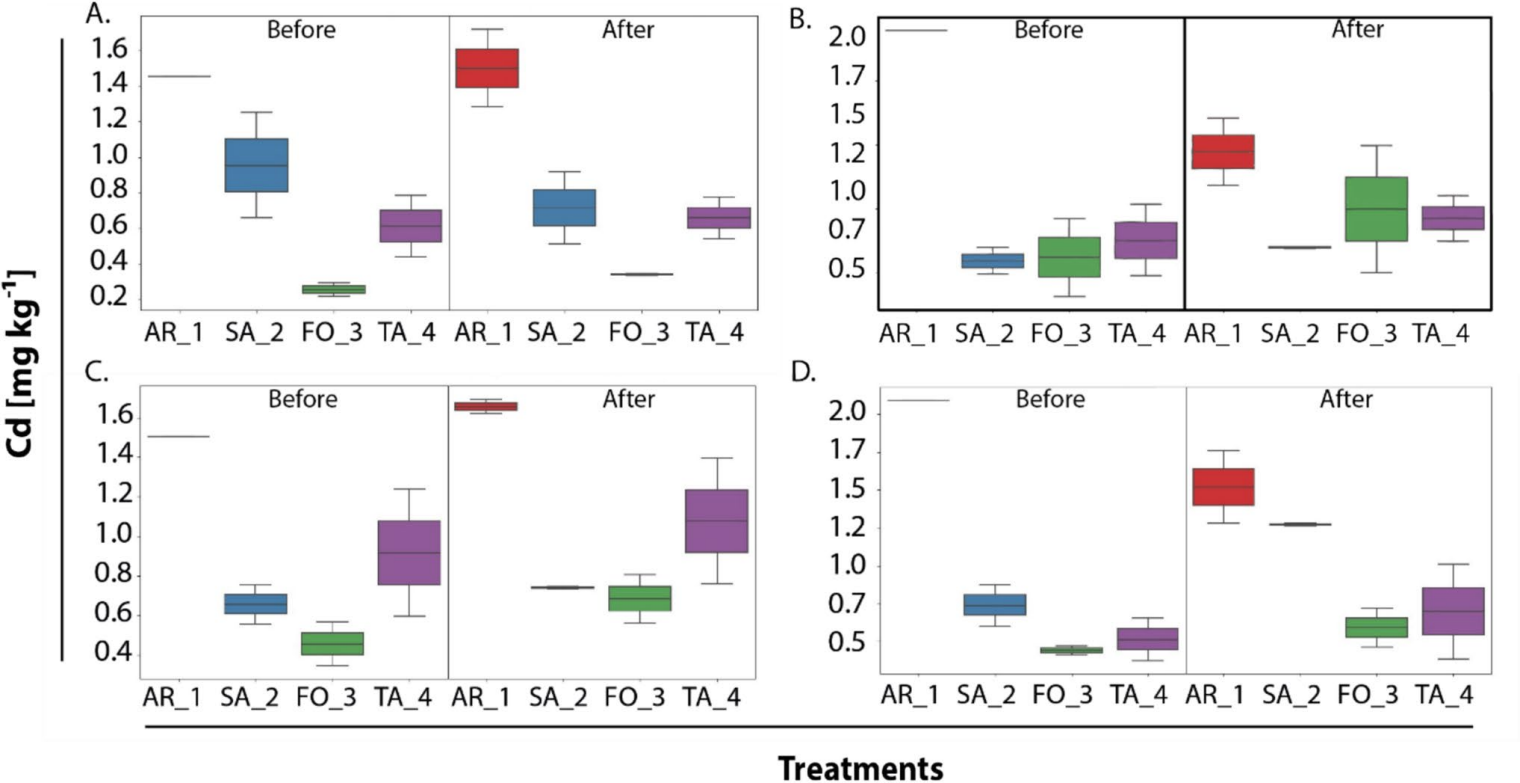
Boxplot of soil Cd in selected samples, before and after application of the treatments: **A** Treatment 3: 12.5% CdtB, 12.5% zeolite and 75% Agrocacao **B** Treatment 5: 25% CdtB, 75% Agrocacao **C** Treatment 7: 25% CdtB, 50% zeolite and 25% Agrocacao. **D** Treatment 8: 50% CdtB and 50% Agrocacao, on cacao farm soils in the following locations: Arauquita (AR_1), Saravena (SA_2), Fortul (FO_3) and Tame (TA_4). Abbreviations: ‘Before’ = Cd in samples before applying the treatments; and ‘After’ = Cd in samples after soil treatment applications. Vertical bars correspond to the error bar (n = 2). The one-sample t-test showed significant differences (p < 0.5)

The concentration of pseudo-total soil Cd was determined before (t_0_) and after (t_1_) the application of the treatments, Fig. 1 shows the Cd values determined at the two application times. The highest recorded Cd value is 2.39 mg kg⁻^1^. This was recorded in AR_1, prior to application of the treatments T7, T9 and T11 (t_0_). The lowest Cd value is 0.31 mg kg⁻^1^. This value occurs in farms FO_3 and TA_4, after treatments T3, T5 and T7 (t_1_). Figure 1 shows the combined mean at t_0_ was 0.75, while at t_1_ it was 0.94. The maximum value found at t_0_ was 2.39 and at t_1_ it was 1.75, while the minimum value found at t_0_ was 0.21 and at t_1_ it was 0.30. In all cases, the statistical significance level of the multifactorial ANOVA was < 0.5. A boxplot of the Cd determination and outliers in soils is shown in Fig. 2. The figure compares the Cd content between farms (boxes) and municipalities (panels). Treatment T9 (no application) serves as a control (not shown in the figure).

Figure 2A shows that, before treatments applications, higher Cd content was observed in AR_1 and SA_2 with 1.5 and 1.3 mg kg^−1^, respectively. The lowest was FO_3 with 0.3 mg kg^−1^. However, after the treatment 3 (12.5% CdtB, 12.5% zeolite, 75% Agrocacao), the Cd concentration at farm AR_1 increased to 1.7 mg kg^−1^, whereas SA_2 decreased to 0.9 mg kg^−1^. Figure 2B, corresponding to treatment 5 (25% CdtB, 75% Agrocacao), shows an initial Cd content in AR_1 with 2.5 mg kg^−1^ as the highest. Interestingly, after treatment application, the same farm decrease to 1.5 mg kg^−1^ Cd, whereas FO_3 increase to 1.25 mg kg^−1^. Figure 2C, that corresponds to the treatment 7 (25% CdtB, 50% zeolite, 25% Agrocacao), shows a high Cd content in AR_1 before treatment, with 1.5 mg kg^−1^, and increase after the application of the treatment to 1.7 mg kg^−1^. Cd content slight increase also in other municipalities assessed. Moreover, in Fig. 2D, before treatment 8 (T8: 50% CdtB, 50% Agrocacao), the highest Cd content again is observed in farm AR_1 with 2.5 mg kg^−1^ on average, whereas the lower is found in FO_3 with 0.4 mg kg^−1^. In contrast, after the application of the treatment, the farm AR_1 decrease to 1.75 mg kg^−1^ and SA_2 increase to 1.25 mg kg^−1^ Cd on average.

It is noted that farm AR_1 with the treatment T8 consistently shows the highest Cd levels before treatment (2.4 mg kg⁻^1^) but also the most significant reductions post-treatment (1.3—1.75 mg kg⁻^1^), likely due to the high CdtB (50%) in T8. Besides, the farm FO_3 with the treatment T7 shows increase in Cd, in Fig. 2C and D, with values, for instance from 0.6 to 0.9 mg kg⁻^1^ in Fig. 2C, possibly due to zeolite (50%), known for Cd adsorption. For other farms such as in SA_2 with treatment T3, there was a decrease in Cd post-treatment with an initial content of 0.9 and a final content of 0.7 mg kg^−1^, suggesting that Agrocacao alone is unable to regulate available Cd, not outweighing the effects of CdtB combined.

Interestingly, in terms of Cd availability, at the beginning of the experiment, the available Cd, on average, was 0.05 mg kg^−1^ Cd, in the four farms. At the end of the applications, a decreasing pattern of the available fraction of Cd was noted, with values of 0.02, 0.027, 0.03 and 0.07 mg kg^−1^, for Arauquita, Saravena, Fortul and Tame, respectively. The mean of available soil Cd at the end of the experiment was 0.036 mg kg^−1^ Cd. This is lower than 0.05, thus the alternative hypothesis of Cd decrease is supported with the one-sample t-test (0.06, p-value 0.1624), however, the variability is high. Cd in leaves also shows a tendency to decrease when comparing the initial average Cd content of 10.03 mg kg^−1^, with the values 8.02, 8.53, 9.00 and 9.27 mg kg^−1^ Cd found at the end of the experiment for Arauquita, Saravena, Fortul and Tame, respectively.

Regardless the farms and treatments, the variability across the soil samples studied here suggests differences in sampling conditions (e.g., seasons, micro-climate variations and specific surrounding environmental conditions of each farm), but the pattern of TA_4’s high initial Cd and subsequent reduction is consistent.

A key issue to discuss is the initial heterogeneity in cadmium levels between farms in Arauca. Figure 1 confirms that Cd levels in Arauca’s cacao farms vary significantly, likely influenced by both geogenic (sedimentation) and anthropogenic (manure fertilisers) sources as reported recently (Bravo et al. 2024a, b; López-Zuleta et al. 2025). Farms such as AR_1, SA_2, tend to present higher levels of pseudo-total cadmium, compared to FO_3, which consistently shows the lowest concentrations. Thus, this basal variability suggests the influence of site-specific changes that drives different natural accumulation of cadmium in the soil to each farm. This has been observed in previous studies in Colombia in the departments of Antioquia (Jaramillo-Mazo et al. 2024) and Santander (Adarme-Duran et al. 2024).

The variability of the data within each farm is also an important element of the analysis. Some farms such as in Tame (TA_4) exhibit a high variability in cadmium concentration both before and after the application of the treatments. This may reflect a non-homogeneous distribution of the element in the soil of this farm or a greater sensitivity to environmental conditions or to the application of the treatments themselves. In contrast, other farms such as in Saravena (SA_2), show a greater homogenization of cadmium concentration after the intervention.

Furthermore, when considering the effect of the treatments, no uniform response is identified in all farms, rather, cadmium mobility and concentration seem to be conditioned by the specific characteristics of each site. For example, treatment T7 (Fig. 2C) shows a slight overall increase in cadmium concentration across all farms, which could indicate a mechanism of action of the treatment that favours the mobilization or addition of cadmium to the soil. However, the magnitude of this increase and the subsequent evolution differ between farms suggesting that other factors could be modulating this response.

In this regards, critical soil parameters could also help to understand better the effect of CdtB in the immobilization process. Thus, further studies in these farms should include soil physicochemical parameters such as pH, texture, cation exchange capacity, organic matter, Ca/Mg content, and electrical conductivity that may influence Cd mobility from soil to the cacao plant, as shown in other studies (Gil et al. 2022).

Sedimentation likely contributes to the high baseline Cd in Arauquita and Saravena, as farms closer to the Arauca River experience Cd deposition from alluvial sediments during flooding. The slight Cd increases in AR_1 and SA_2 post-treatment suggests that the treatments with CdtB may favour in soil formation of geostable Cd-compounds, since less available Cd was found at those farms.

The temporal dynamics of cadmium concentration also varies between farms and in response to treatments. These temporal differences highlight the complexity of the processes that drives cadmium behaviour in soil and how these processes can be modulated by farm-specific characteristics and by the interventions applied. The T8 treatment (Fig. 2D) seems to induce a general increase in pseudo-total cadmium concentrations in all farms, although farm AR_1, despite having the highest cadmium content, shows a greater dispersion of data after application, suggesting a non-uniform response. Farm SA_2 experiences a noticeable increase in concentration, while TA_4, with low initial levels, also shows an increase after T8 application.

**Fig. 3:**
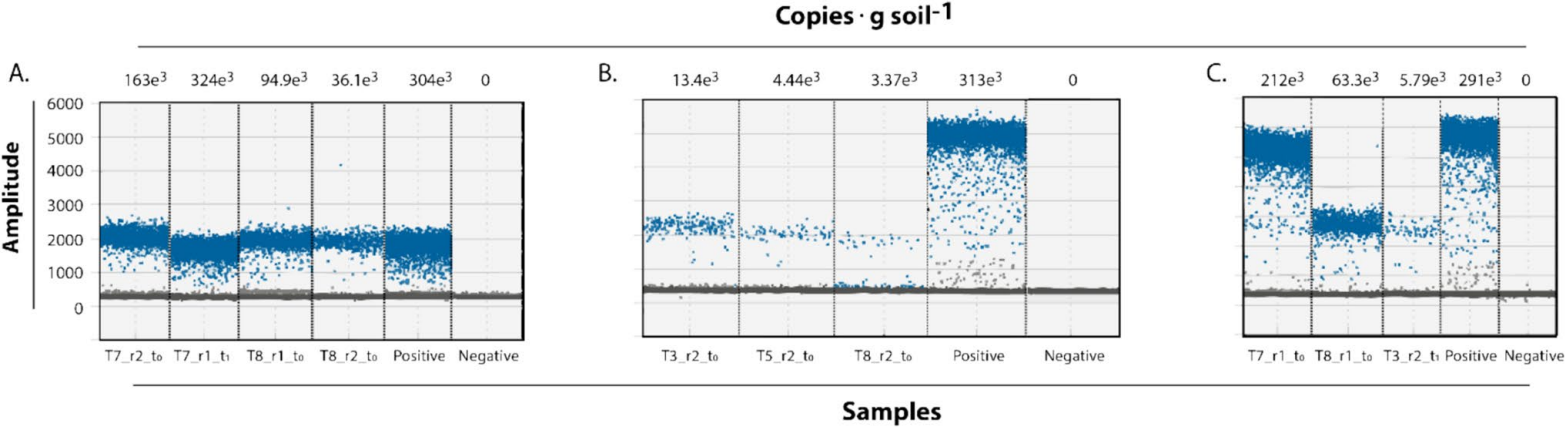
Scatter plots of ddPCR showing the amplification and quantification of number of copies of the gene *cadA* in soil samples from three cacao farms **A** corresponds to the farm SA_2; **B** to the farm FO_3 and **C.** to the farm TA_4. Each scatter plot shows the FAM fluorescence amplitude of the droplets produced during the reaction for the soil treatments. Positive and negative controls were included to validate gene detection. Each droplet is represented with the blue dots, reflecting the presence of the gene

The treatment that showed a trending positive effect for possible immobilization of cadmium is treatment T8 (50% CdtB and 50% Agrocacao; Fig. 2D) followed by T5 (25% CdtB and 75% Agrocacao; Fig. 3B). On the contrary, the treatment that did not show the expected increase of cadmium in the soil was T3 (12, 5% CdtB, 12.5% zeolite and 75% Agrocacao; Fig. 3A) revealing that the effect of zeolite as a chelating agent was not significant in the immobilization of the element. This observation is in line with that reported in Ecuador (Chavez et al. 2015), where zeolite addition did not affect pH of cadmium levels in soils. Similarly, a previous study in Colombia (Quiroga-Mateus et al. 2022), suggests that zeolite is more related to cadmium depletion in soils.

Comparing these results with other studies in Arauca, a mean soil Cd of 1.16 mg kg⁻^1^ was reported with a north–south gradient (higher near the Arauca River, and up to 10.03 mg kg⁻^1^ in soil litter) due to sedimentation (Bravo and Benavides-Erazo 2020). Thus, this study (0.2–1.6 mg kg⁻^1^) align with this range, with TA_4’s higher values suggesting proximity to riverine sedimentation zones (Gonzalez-Orozco et al. 2023; Bravo et al. 2024a, b).

Additionally, in another department, Santander, the most important cacao producing region in the country, the soil Cd averages is 2.76 mg kg⁻^1^, driven by geogenic sources mainly by sedimentary rocks (Bravo et al. 2021) and alluvial deposits, higher than Arauca but consistent with sedimentation as a key driver (Bravo et al., 2026), as in the municipalities studied here.

At regional context, in Ecuador and Peru, both major cacao producer countries, maximum Cd in cacao soils are in 6.9 and 0.87 mg kg⁻^1^, respectively (Argüello et al. 2019; Thomas et al. 2023), far exceeding Araucan soil, which is attributed to alluvial deposits and parent material (Bravo et al. 2024a, b). Arauca’s *Typic Endoaquepts* soils, with low clay content, likely enhance Cd mobility from sedimentation, as seen in Arauquita’s and Saravena’s high initial levels.

The fact that the concentration of cadmium in the soil increases after the application of the treatments does not necessarily imply that more cadmium has been incorporated into the system, but that the metal might be trapped in geostable forms not available to the plant, as suggested in this study. It is possible that the increase in the concentration of pseudo total cadmium in soils is due to the interaction of the CdtB. Bacteria promote mechanisms such as biosorption where the bioaugmented populations binds to the metal ions through structural and functional components.

Not many studies assess the effect of chemical-like fertilisers as Cd input in the cacao system (López-Zuleta et al. 2025). Here, Agrocacao (with less than 0.06 mg kg^−1^ Cd) improved the retention of the metal in the soil through interactions with organic matter, and CdtB in the treatments assessed in Arauca, influencing cadmium content after incorporation into the soil.

Tested treatments may not be uniformly effective in all regions due to site-specific soil characteristics such as chemical composition, texture, structure, organic content and native microbiota, which determine the soil’s ability to retain and mobilize cadmium. In acidic soils such as in Arauca (mean pH value in cacao soils is 5.25; León-Moreno et al. 2019; Bravo et al. 2024a, b), cadmium should be more soluble and therefore more bioavailable, which could affect the efficacy of certain treatments (Gramlich et al. 2017; Gil et al. 2022). Nonetheless, despite all physical–chemical features, other factors such as the soil the temperature and indigenous microbiota activity may play an important role and vary between farms, suggesting the need to tailor remediation strategies to very local conditions to ensure average positive results.

### Detection of *cadA* gene

The *cadA* gene is involved in the antiport process of Cd at the cytoplasmic stage in bioweathering of Cd induced by CdtB (Bravo and Braissant 2022). Therefore, the use of this gene amplification was a first step to understand the impact of bioaugmentation of the CdtB from the applied product. However, as the ddPCR data were derived from composite samples in this study, differences among treatments and times could only be interpreted in terms of indicative patterns in the exploratory trends found here. Figure 3 shows the results of the amplifications and quantification of copies of the *cadA* gene related to cadmium immobilization. It contains three panels (Fig. 3A, B, and C) showing scatter plots of gene amplification (y-axis: Amp, in arbitrary units) for different samples.

As shown in the farm SA_2 (Fig. 3A) the amplification of the *cadA* gene across different samples is highly variable, ranging from 100 to 280,000 unities, with an average of 154.5 × 10^3^ copies g soil^−1^ of the *cadA* gene. The positive control is high, with 304 × 10^3^ copies g soil^−1^ and a dense scatter plot around 2000–3000 Amp units, indicating successful amplification. The negative control shows no copies (close to 0 copies g soil^−1^) and minimal amplification, as expected. The treatment T7_r1_t_1_ (324 × 10^3^ copies g soil^−1^) shows the highest amplification among the treatments, with a dense scatter plot around 2000 Amp units, suggesting effective *cadA* gene expression at time t1. Moreover, T7_r2_t_0_ (163 × 10^3^ copies g soil^−1^) has moderate amplification, with a slightly lower density around 1500–2000 Amp units. On the contrary, T8_r1_t_0_ (94.9 × 10^3^ copies g soil^−1^) and T8_r2_t_0_ (36.1 × 10^3^ copies g soil^−1^) show lower copy numbers and sparser scatter plots, indicating weaker *cadA* gene amplification compared to T7.

Likewise, the cadA gene was detected in low concentrations in baseline samples of soils of FO_3 (Fig. 3B), compared to its amplification in treatments T3, T5 (25% CdtB), and T8, alongside controls. The positive control again shows strong amplification (313 × 10^3^ copies g soil^−1^) with a dense scatter plot around 4000–5000 Amp units, higher than in Fig. 3A, indicating robust *cadA* gene detection. The negative control shows no amplification (0 copies g soil^−1^). Interestingly, the treatment T3_r2_t_0_ (13.4 × 10^3^ copies g soil^−1^) has the highest copy number among the treatments but is still very low compared to the positive control. Also, the treatments T5_r2_t_0_ (4.44 × 10^3^ copies g soil^−1^) and T8_r2_t_0_ (3.37 × 10^3^ copies g soil^−1^) show even lower copy numbers and minimal amplification, with scattered points mostly below 500 Amp units, suggesting very weak *cadA* gene amplification in these samples.

Farm AR_1 (Fig. 3C) shows a good amplification of the *cadA* gene. The positive control shows strong amplification (291 × 10^3^ copies g soil^−1^) with a dense scatter plot around 4000–5000 Amp units, consistent with Fig. 3B. The negative control again shows no amplification (copies g soil^−1^). For the treatments, T3_r2_t_1_ (212 × 10^3^ copies g soil^−1^) shows a significant increase in copy number compared to T3_r2_t_0_ in Panel B (13.4 × 10^3^ copies g soil^−1^), with a denser scatter plot around 3000–4000 Amp units, indicating that *cadA* gene amplification increased over time. Treatments T8_r1_t_0_ (63.3 × 10^3^ copies g soil^−1^) and T8_r2_t_0_ (5.79 × 10^3^ copies g soil^−1^) show low copy numbers, with the latter having the lowest amplification in this panel, consistent with its weak performance in Panels A and B. Therefore, high copy numbers are likely to be related to major gene amplification, that could be occur due to high CdtB population density.

The *cadA* gene encodes a cadmium-transporting ATPase, which plays a role in Cd tolerance and immobilization in soil by facilitating Cd sequestration or efflux in microorganisms, as well as other genes related in the same operon, such as *cadD* (Naz et al. 2005) and *zntA* (Naz et al. 2005; Singh et al. 2020), all important in the bioleaching and biotransformation metabolic pathways (Bravo and Braissant 2022). In the context of cacao farms in Arauquita, Fortul, Saravena, and Tame, successful bioaugmenting of *P. chlororaphis* as shown through amplification of the *cadA* gene, is a first step in being about to understand if this could enhance Cd immobilization, reducing its bioavailability and thus uptake by cacao plants.

The ddPCR technique used for the analysis here offers high sensitivity and precision that allows a detailed characterization of the presence and abundance of this gene in the soil microbial communities. The results reveal significant variability in the presence and abundance of the *cadA* gene among the farms studied, with some farms detecting this gene in various concentrations even before the application of treatments, suggesting a heterogeneous distribution of CdtB, which could be related to soil factors such as texture, pH, nutrient availability and presence of indigenous microbiota. Despite this, the treatment T7_r1_t_1_ (324 × 10^3^ copies g soil^−1^) shows interesting results in amplification particularly after the application of the treatment (Fig. 3A). In this soil, the high zeolite content likely enhances Cd immobilization by adsorbing Cd ions, while CdtB, supports the increase in microbial activity, leading to increased *cadA* gene amplification. This suggests that T7 has a positive trend with additions of CdtB to the soil. In contrast, T3 shows a time-dependent increase in *cadA* gene amplification after the application. The lower zeolite and CdtB proportions, combined with a high chemical fertilizer component, may initially limit microbial activity or *cadA* gene expression. However, over time (passing from t_0_ to t_1_), the microbial community may adapt, leading to increased *cadA* gene expression and better Cd immobilization. Likewise, T8 consistently shows that the absence of zeolite, low CdtB charge and high chemical fertilizer content may suppress microbial activity or *cadA* gene abundance, leading to poor Cd immobilization.

In addition to spatial differences in *cadA* gene abundance, variations in the response of microbial communities to treatments are also observed, in some cases the application of certain treatments is associated with an increase in gene copy number suggesting a possible stimulation of CdtB activity, however, this varies between treatments and locations indicating a complex interaction between treatment composition, soil characteristics and microbial community dynamics.

### Metabolic activity associated with CdtB

**Fig. 4:**
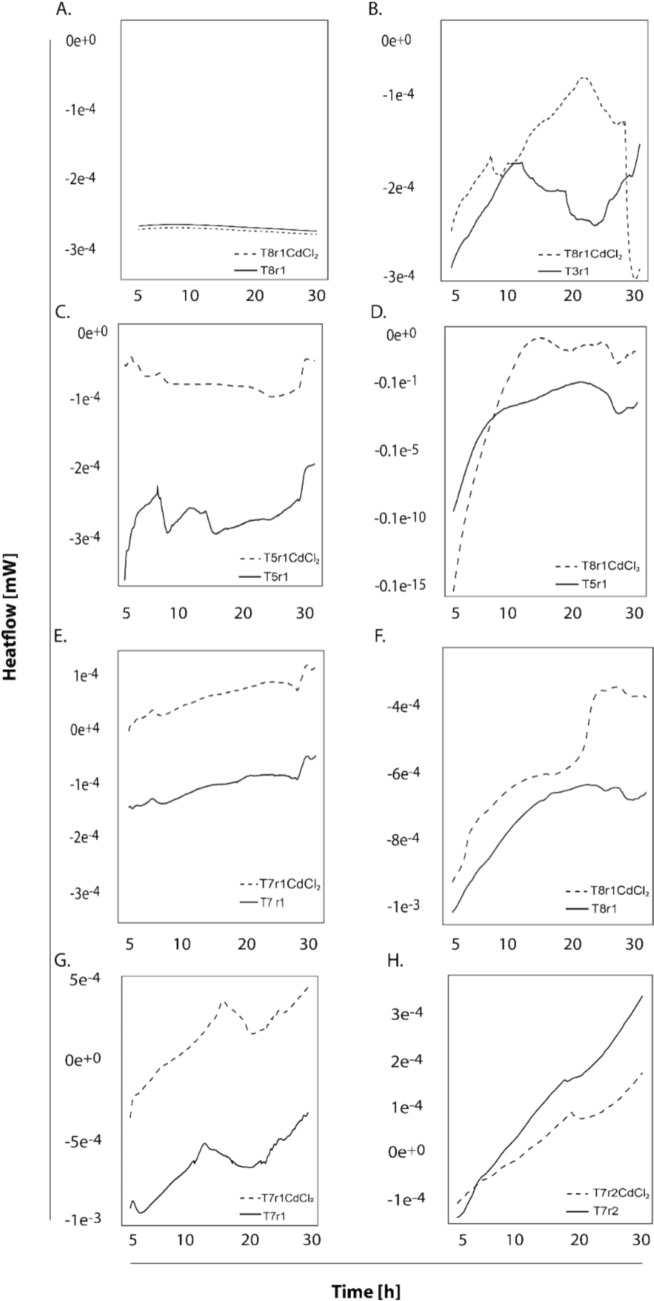
Thermograms showing the heat-flow over 30 h of thermal monitoring of soil samples assessed in Arauca. The comparison is carried out between soil samples with (dashed lines) or without (solid lines) Cd spiking in the vessel. **A** AR_1 (t_0_); **B** AR_1 (t_1_), **C** SA_2 (t_0_); **D** SA_2 (t_1_); **E** FO_3 (t_0_); **F** FO_3 (t_1_); **G** TA_4 (t_0_) and **H** TA_4 (t_1_)

The IMC technique allowed a preliminary assessment of the effect of the application of bioremediation treatments on the metabolic activity of the microbial communities present in cacao farm soils. The comparison between the thermograms obtained before (t_0_) and after (t_1_) the application of the treatments and the combined sources of the treatments is key to understanding these changes. Figure 4 shows the results of the microbial metabolic assessment of the soil samples studied with IMC. The raw data of these thermograms can be found in Table S1. Figure 4 consists of eight plots (A to H) that depict the heat-flow measured using the TAM Air, for soil samples over a 30-h period. The samples are either untreated (labelled as T5r1 or T7r1) or treated with an amendment of CdCl₂ (labelled as T5r1CdCl₂ or T7r1CdCl₂). The x-axis represents time in hours (0 to 30 h), and the y-axis represents the heat-flow.

In Fig. 4A, both samples show minimal heat-flow, remaining close to 0 mW throughout the 30 h, with the T5r1CdCl₂ (dashed line) and T5r1 (solid line) showing almost identical, flat profiles. Figure 4B shows that T3r1 (solid line) remains near 0 mW, while T8r1CdCl₂ (dashed line) shows a significant peak around 15 h, reaching approximately −2 e^−2^ mW, followed by a decline. In Fig. 4C the sample T5r1 (solid line) shows a slight increase in heat-flow, peaking around −1e^-4^ mW at 10 h, while T5r1CdCl₂ (dashed line) remains flat near 0 mW. Besides, Fig. 4D with sample T5r1 (solid line) remains near 0 mW, while T8r1CdCl₂ (dashed line) shows a sharp increase, peaking at −0.1e^-15^ mW around 15 h, then gradually declining. In Fig. 4E the sample T7r1 (solid line) shows a small peak around 1e^-4^ mW at 10 h, while T7r1CdCl₂ (dashed line) remains flat near 0 mW. Figure 4F compares T8r1 (solid line) that remains near 0 mW, while T8r1CdCl₂ (dashed line) shows a dramatic peak at -8e^-3^ mW around 15 h, followed by a decline. Figure 4G showed T7r1 (solid line) with a peak around -1e^-3^ mW at 10 h, while T7r1CdCl₂ (dashed line) remains relatively flat, with minor fluctuations near 0 mW. In contrast to Fig. 4G, H shows T7r2 (solid line) with a steady increase, reaching 2 e^-4^ mW by 30 h, while T7r2CdCl₂ (dashed line) shows a slower increase, reaching 1e^-4^ mW by 30 h.

As explained earlier, the *cadA* gene codes for a cadmium resistance protein, often found in CdtB, which helps detoxify cadmium ions (Cd^2^⁺) by pumping it out of the cell, reducing its toxic effects.

In regard to the relationship between the *cadA* gene amplified by ddPCR and metabolic ratios characterised by IMC, assume that *cadA* gene presence might increase when the CdtB is bioaugmented in soils even without addition of soluble Cd. In Fig. 4B, the significant peak in T8r1CdCl₂ suggests that CdCl₂ stimulates microbial activity, possibly due to stress response or the activation of *CadA*-like mechanisms that increase metabolic energy expenditure to detoxify cadmium. T3r1, without CdCl₂, shows no such response, indicating baseline microbial activity. In Fig. 4C, T5r1 shows a small increase in heat-flow, possibly due to natural microbial activity, while T5r1CdCl₂ remains flat, suggesting that cadmium inhibits microbial activity in this sample. The lack of a *CadA*-driven response may indicate that cadmium toxicity overpowers any resistance mechanisms. In Fig. 4D, T8r1CdCl₂ shows a sharp increase in heat-flow, indicating a strong microbial response to CdCl₂, likely due to *CadA*-mediated detoxification processes that increase metabolic activity. T5r1, without CdCl₂, remains inactive, serving as a control. In Fig. 4E T7r1 shows a small peak, reflecting baseline microbial activity, while T7r1CdCl₂ remains flat, suggesting cadmium toxicity suppresses activity, potentially due to insufficient *CadA* amplification (among other genes related to immobilisation) or activity in this sample. On the contrary, in Fig. 4F, the dramatic peak in T8r1CdCl₂ indicates a robust microbial response to CdCl₂, likely driven by *CadA* gene or similar cadmium resistance mechanisms, leading to increased heat-flow as energy is expended for detoxification. T8r1 remains inactive, showing no such response without CdCl₂. Figure 4G with the treatment T7r1 shows a peak in heat-flow, reflecting natural microbial activity, while T7r1CdCl₂ remains flat, suggesting cadmium toxicity suppresses activity, possibly due to limited *CadA*-mediated resistance. And in Fig. 4H, both, T7r2 and T7r2CdCl₂ show a gradual increase in heat-flow, with T7r2 being higher. This suggests that while cadmium (in T7r2CdCl₂) slightly suppresses microbial activity, the presence of *CadA* gene or other resistance mechanisms may mitigate some of the toxic effects, allowing for a slower increase in activity compared to the untreated T7r2.

The presence of zeolite could modulate the activity of CdtB, possibly by reducing cadmium availability due to its chelating properties. The microbiota could also reduce its activity by not activating energy-requiring mechanisms. (Bravo 2022; Bravo and Braissant 2022).

**Fig. 5:**
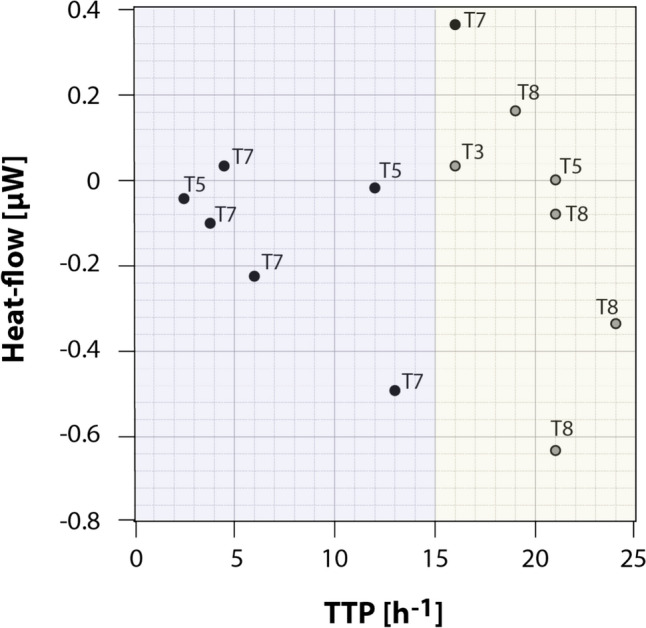
A comparison between the heat-flow and the time to the peak (TTP) is shown for treatments that produced significant heat peaks due to bacterial metabolic activity, obtained by IMC, at t_0_ (black dots) and t_1_ (grey dots). The violet background represents fast metabolic processes recorded, whereas the yellow background represents the slow metabolic processes associated to Cd input. Abbreviations: T3, 5, 7 and 8 corresponds to the treatments assessed

Figure 5 shows a comparison of the temporal dynamics of microbial metabolic activity in treated and untreated soils, revealing significant differences between the rate and pattern of metabolic response. The chart shows black dots that represents the basal metabolic activity of the soil without intervention reflecting the endogenous activity of the indigenous microbiota, while the grey dots illustrate the response of the soil and microbiota under treatment application.

A key observation is the clear separation between the two groups of dots suggesting an important modulation in microbial metabolism in treated compared to untreated soils. The black dots are predominantly concentrated in the time range of 2 to 14 h. This early clustering indicates a rapid metabolic activation (highlighted in violet background in Fig. 5) of the bacteria in untreated soils. This response could be interpreted as an immediate reaction to the stress imposed by the presence of cadmium. Native microorganisms already adapted to the soil conditions may rapidly activate stress response mechanisms such as the expression of heavy metal resistance genes, the production of chelating substances or the alteration of metabolic pathways to mitigate toxic effects of cadmium (Bravo et al. 2015, 2018).

In contrast, treated soils (grey dots) exhibit a shift of metabolic activity to a later time range, mainly concentrated between 15 and 24 h, showing slow metabolic flux (highlighted in yellow background in Fig. 5). This delay in the onset of metabolic activity suggests that microbial communities in treated soils require a longer period of time to initiate their significant metabolic response, this delay could be attributed to several related factors among which the following stand out: i. heterogeneity of soils that require time for microorganisms to explore, find suitable niches and finally access the available resources of those soils; and ii. symbiotic, competitive or antagonistic interactions between native and introduced microbial populations that may modulate the speed of metabolic response. This suggests that bioremediation induces significant changes in the structure and function of soil microbial communities.

### Cadmium stress and microbial activity

Studies have shown that cadmium can significantly alter soil microbial activity due to its toxicity. For instance, a previous study (Renella et al. 2006) found that cadmium exposure typically reduces microbial respiration and metabolic activity in soil, as it disrupts cellular processes. This aligns with plots A, C, E, and G of Fig. 3, where CdCl₂-treated samples (e.g., T5r1CdCl₂, T7r1CdCl₂) show suppressed heat-flow compared to untreated samples, suggesting cadmium toxicity overwhelms microbial resistance mechanisms in these soils.

### *CadA*-mediated tolerance

Previous research (Nies 1999) highlights that *CadA*, the P-type ATPase, is a common cadmium resistance mechanism in bacteria such as *Staphylococcus aureus* and *Pseudomonas* species, which are often found in soil. *CadA* pumps Cd^2^⁺ out of the cell, requiring energy (ATP), which could explain the increased heat-flow in plots B, D, and F (e.g., T8r1CdCl₂) in Fig. 2, where CdCl₂-treated samples show significant peaks. The energy expenditure for cadmium detoxification likely increases microbial metabolic rates, resulting in higher heat-flow. The increase in *cadA* gene amplification from t_0_ to t_1_ in T3 (13.4 to 212 copies μL⁻^1^) is consistent with literature showing that microbial communities adapt to soil amendments over time. Studies on biochar-amended soils often report a lag phase in *cadA* gene expression, followed by a sharp increase after 30–60 days as microbial populations stabilize. This aligns with the increase in T3, suggesting that even low zeolite and CdtB levels can eventually support *CadA*-mediated Cd immobilization.

In this research, the aim of using the molecular analysis was to assess bacterial bioaugmentation, i.e., the abundance of the CdtB population in the soil after application of the treatments, measured indirectly by the variation in copies g soil⁻^1^ of the *cadA* gene found between treatments. In this context, the increase in *cadA* gene abundance is interpreted as an indicator of the colonisation success, of *P. chlororaphis*, but not as a direct measurement of Cd immobilisation.

To demonstrate actual immobilisation, it is necessary to apply additional geochemical analyses, such as sequential extractions or chemical speciation of Cd, to verify the change of Cd from bioavailable to less mobile fractions such as carbonates, oxides, residuals or recalcitrant fractions (Rinklebe and Shaheen 2014; Cifuentes et al. 2025).

### Variability in soil microbial responses

A previous study (Hattori 1992) notes that soil microbial responses to cadmium vary depending on soil type, microbial community composition, and the presence of tolerance genes. This variability is evident across the plots. For example, the result of the present study with the treatment T8r1CdCl₂ (plots B, D, F in Fig. 3) consistently shows a strong response to CdCl₂, suggesting a microbial community with probable active *CadA* or similar mechanisms, while T5r1CdCl₂ and T7r1CdCl₂ (plots A, C, E, G of Fig. 3) show suppression, indicating a lack of sufficient tolerance.

The variability in heat-flow responses across the plots suggests that the microbial communities in these soil samples differ in their cadmium tolerance capabilities, likely influenced by the presence and expression of *cadA* or similar genes. Samples like T8r1CdCl₂, which show significant heat-flow peaks, likely contain microbial populations with active cadmium tolerance mechanisms, leading to increased metabolic activity as they detoxify Cd^2^⁺. Conversely, samples like T5r1CdCl₂ and T7r1CdCl₂, which show suppressed activity, may lack sufficient resistance, resulting in cadmium toxicity inhibiting microbial metabolism.

### Microbial adaptation

A paper by (Zhang et al. 2022), suggests that exposure to cadmium can lead to long term microbial adaptation, where communities develop tolerance mechanisms over time. Plot H of Fig. 3, where T7r2CdCl₂ shows a gradual increase in heat-flow (though lower than T7r2), may indicate an adaptive response, possibly due to the presence of *cadA* gene or other mechanisms mitigating cadmium toxicity over the 30-h period.

It is clear that this is a short-term experiment to understand the effect of soil CdtB bioaugmentation in decreasing Cd in cacao crop being part of a larger study (Bravo et al. 2026). Future research in Arauquita, Fortul, Saravena, and Tame could focus on correlating *cadA* gene copy numbers with Cd levels in cacao beans and soil, as well as identifying the specific microbial taxa driving *cadA* gene expression in both native populations and the bioaugmented CdtB bioproduct population. Incorporating metagenomic analysis, could reveal how treatments like T7, T8 shape CdtB communities.

Moreover, to understand the suggested mechanisms of Cd immobilisation mentioned here, we propose to i. quantify Cd bioavailability using DGT methods; ii. determine Cd concentration in cacao beans to link soil data to plant accumulation (Bravo et al. 2024a, b); iii. characterise soil physicochemical properties and amendment compositions (Gil et al. 2022); iv. validate ddPCR methodology with efficiency and specificity tests; and v. quantify the persistence of CdtB strains in treated soils combining molecular with culture-based approaches to enhance the microbial composition of cacao soil CdtB communities in Arauca. This implies a microcosm assay using *P. chlororaphis* as the main population driving soil Cd sequestration through geochemical stabilisation.

## Conclusions

The *cadA* gene amplification with ddPCR and the recorded activity indicating possible immobilisation of Cd with IMC, were used to analyse the effect of CdtB bioaugmentation with *P. chlororaphis* in cacao soils, as a novel approach to understand bioremediation of Cd accumulation in cacao in Colombia. Although these methodologies were used for exploratory analyses, the results obtained underline the importance of considering inter-farm variability when assessing the impact of treatments. Even though a single uniform response was not found with the applied interventions, this study furthered our understanding of the effectiveness and direction of change in cadmium levels that could be strongly influenced by the baseline characteristics of each farm and by the specific interaction between the bioremediation treatment and local conditions. These findings also highlight the need for farm- and soil-specific analyses when implementing cadmium management strategies in cacao crops. Future research could focus on identifying the soil, geological or microbiological factors that underlie these differences in response to treatments. This could help to develop more efficient remediation or management strategies tailored to local conditions. Long-term monitoring is necessary. Changes in soil microbiological properties, such as the development of microbial communities adapted to contaminated conditions, require time to stabilise and show a measurable effect on cadmium variation over time, as shown with the calorimetric assay. The effectiveness of the CdtB *P. chlororaphis* applied with Agrocacao (T7 and T8), may depend on factors such as adaptation to the local environment, with high soil Cd contents and the interaction with native microbiota and persistence over time. Therefore, while the first step on this pilot scale field applications has been carried out here, forthcoming studies in long-term applications may elucidate the real impact of bioremediation in cacao.

## Significance of the work

The research addresses the contribution of cadmium-tolerant bacteria (CdtB) in immobilizing cadmium in cacao soils as a strategy to prevent its translocation to the plant and, consequently, to the beans.

The importance of this research lies in its contribution to solving a critical issue for regions such as Arauca, one of the main fine cacao-producing areas in Colombia. The presence of cadmium in the beans has significantly limited export opportunities for the product, affecting the socio-economic development of local farmers. Thus, the research focuses on the use of bacteria with the ability to immobilize cadmium in the soil, delving into the mechanisms of in situ microbial bioremediation, an environmentally sustainable strategy. To assess the effectiveness of the process, advanced methodologies such as isothermal calorimetry (IMC) were applied to analyze the metabolic activity of microorganisms, as well as, the detection of the *CadA* gene associated with cadmium tolerance. These approaches not only validated the functionality of the process but also provided valuable information on microbial gene expression in response to cadmium.

The findings are relevant for the development of biotechnological products applicable to agricultural production, as they promote the resilience of cropping systems to abiotic contamination, ensuring the economic and social sustainability of the cacao value chain in affected regions. Henceforth, we believe that the journal 3 BIOTECH is an ideal venue for the publication of this work, as it addresses topics related to environmental, microbial and agricultural biotechnology, all of which are present in this research. In addition, it promotes applied studies with a focus on sustainability, food safety and the development of innovative bioprocesses, in line with the aims of our study.

## Data Availability

Since there are economic and social concerns surrounding cadmium in cacao in Colombia, the data included in this study are not available. However, they may be provided via a written request to the office of intellectual property of AGROSAVIA in Colombia, copying the corresponding author of this study.
